# Association of Serum Irisin with Metabolic Syndrome in Obese Chinese Adults

**DOI:** 10.1371/journal.pone.0094235

**Published:** 2014-04-07

**Authors:** Bing Yan, Xiulin Shi, Huijie Zhang, Lingling Pan, Zhimin Ma, Suhuan Liu, Yongwen Liu, Xiaoying Li, Shuyu Yang, Zhibin Li

**Affiliations:** 1 Xiamen Diabetes Institute, Xiamen, China; 2 Department of Endocrinology and Diabetes, The First Affiliated Hospital, Xiamen University, Xiamen, China; 3 Shanghai Institute of Endocrinology and Metabolism, Rui-Jin Hospital, School of Medicine, Shanghai Jiaotong University, Shanghai, China; 4 Department of Endocrinology, The Second Affiliated Hospital of Soochow University, Suzhou, China; 5 Department of Nursing, The First Affiliated Hospital, Xiamen University, Xiamen, China; 6 Epidemiology Research Unit, The First Affiliated Hospital, Xiamen University, Xiamen, China; University of Central Greece, Greece

## Abstract

Irisin, a recently identified novel myokine, drives brown-fat-like conversion of white adipose tissues and has been proposed to mediate beneficial effects of exercise on metabolism. Circulating irisin was significantly reduced in type 2 diabetes patients; however, no evidence is available about its association with metabolic syndrome (MetS) and effects of adiposity and muscle mass on circulating irisin have been controversial. Cross-sectional data on socio-demographic, lifestyle, clinical characteristics and serum irisin were collected for 1,115 community-living Chinese adults with central obesity. Associations of serum irisin with MetS (central obesity plus any two of the following four factors (raised blood pressure (BP), raised fasting plasma glucose (FPG), raised triglyceride (TG), and reduced HDL cholesterol) and each component of MetS were analyzed using multivariable logistic regression. Among the 1,115 obese Chinese adults with a mean age of 53.2(±7.2) years, serum irisin levels (log-transformed) were significantly reduced in subjects with MetS and raised FPG than their control groups (p = 0.034 and 0.041, respectively). After adjustment for potential confounders, serum irisin was significantly associated with reduced risks of MetS and raised FPG, with odds ratios (ORs) (95% CI) per standard deviation of log-transformed irisin of 0.796 (0.505–0.959, p = 0.027) and 0.873 (0.764–0.998, p = 0.046), respectively. Associations of irisin with raised BP, raised TG and reduced HDL were not statistically significant ((ORs) (95% CI): 0.733(0.454–1.182, p = 0.202), 0.954(0.838–1.086, p = 0.478) and 1.130(0.980–1.302, p = 0.092), respectively). Stepwise multivariable linear regression analysis showed that fasting insulin, HbA1c and albumin/globulin ratio were negatively associated with serum irisin level with statistical significance (all p-values <0.05) and waist circumference was negatively associated with serum risin with marginally statistical significance (p = 0.055). These results imply that irisin may play an important role in insulin resistance and MetS and should be confirmed in future prospective studies.

## Introduction

Metabolic syndrome (MetS) represents a cluster of atherogenic risk factors including hypertension, insulin resistance, obesity and dyslipidemia, and is now considered as a major public health problem because of its rapidly increasing prevalence worldwide and its association with type 2 diabetes and cardiovascular disease [1]–[3]. Insulin resistance plays an important role in the pathogenesis of MetS although the mechanisms underlying insulin resistance are not fully understood [1]. Physical exercise, as a lifestyle intervention approach, has been consistently shown to be effective in reducing incidence of type 2 diabetes [4], [5] and MetS [6], which made the discovery that physical exercise provokes increases in a number of cytokines from skeletal muscle as a potential mechanism sound plausible.

Irisin, a recently identified novel myokine, is proteolytically processed from the product of the FNDC5 gene prior to being released into the circulation and regulated by PPAR-γ coactivator-1(PGC1)-α [7]. Irisin drives brown-fat-like conversion of white adipose tissues, and has been proposed to mediate the beneficial effects of exercise on metabolism [7]. Circulating irisin was found to be significantly reduced in long-term [8], new onset [9] and undefined [10] type 2 diabetes patients compared with non-diabetic controls, which suggested either the diabetic state itself or the metabolic condition that caused progression to type 2 diabetes is accompanied by lower circulating irisin [11]. However, no evidence is available on whether circulating irisin is involved in metabolic syndrome in adults.

Available evidence about the effect of adiposity on circulating irisin has been controversial. Liu and co-workers found a positive association of irisin with BMI and glucose in non-diabetic subjects but not in diabetes patients, even after adjustment for multiple covariates [8]. The positive association of circulating irisin with BMI has also been found in two other non-diabetic populations [12], [13]. Huh and co-workers further found that muscle mass was the main predictor for circulating irisin [12]. In contrast to these reports above, Moreno-Navarrete et al. found a negative association of circulating irisin with BMI, waist-hip ratio and fat mass in men; although they did find FNDC5 expression in human muscle positively correlated with BMI as well as PGC-1α expression [10]. Therefore, further studies are warranted to address this discrepancy on the effect of adiposity on circulating irisin.

In the present cross-sectional study of 1,115 obese Chinese adults without any previously diagnosed chronic diseases, we aimed to examine the independent effect of circulating irisin on MetS and further determine the association of adiposity with circulating irisin level.

## Materials and Methods

### Subjects

Obese adults were local residents aged 40 years or older living in the Lianqian community, Xiamen, China, and were screened with physical examination from April 2011 to August 2011. Subject sampling, recruitment and evaluation have been described earlier [14]. Briefly, a total of 1,523 subjects with central obesity (waist circumference greater than 90 cm for men and 80 cm for women) were included. Of them, 1,115 (73.2%) subjects with the complete data on the entire examination were left for further analysis.

### Ethics Statement

The study was approved by the Human Research Ethics Committee of the First Affiliated Hospital of Xiamen University (Xiamen, China). Written informed consent was obtained from each participant.

### Measurements

Screening protocol and evaluation criteria were described elsewhere [14]. Staffs participating in this study are doctors and medical students, who received intensive training for epidemiologic screening methods. Data were collected at the community health service centers. The standard questionnaire was used during face-to-face interview to collect socio-demographic status, lifestyle habits (including physical activities using the International Physical Activity Questionnaire –Long form), present and previous health history and medications for each subject. Subjects, who had cancer, current treatment with systemic corticosteroids, biliary obstructive diseases, acute or chronic virus hepatitis, drug-induced liver diseases, total parenteral nutrition, autoimmune hepatitis, Wilson's disease, known hyperthyroidism or hypothyroidism, were excluded.

### Anthropometric measurements

Anthropometric measurements were obtained using standard protocols and techniques. After removal of shoes and heavy clothing, each subject underwent weight, height and waist circumference measurements, using a calibrated scale. Body mass index (BMI) was calculated as weight in kilograms divided by height squared in squared meters as a measure of general obesity. Waist circumference was measured at the midpoint between the inferior costal margin and the superior border of the iliac crest on the mid-axillary line. Body fats were quantified with the HOLOGIC whole body DXA systems (Hologic Inc., Bedford, MA). Arterial blood pressure was measured with a mercury sphygmomanometer after sitting for at least 15 minutes. Blood pressure measurements were taken according to the Joint National Committee VII criteria (JNC VII) [15]. Three readings were taken at 5-min intervals. The mean of the three measurements was recorded.

### Biochemical measurements

75-g oral glucose tolerance test and blood biochemical measurements were conducted for each subject. All blood samples were obtained after 12-hour fasting. Blood and urine samples were refrigerated at −20°C, transferred and tested in the centre laboratory of the First Affiliated Hospital, Xiamen University. Plasma glucose, liver enzyme levels, and serum lipid profiles, including triglyceride (TG), total cholesterol (TC), and high-density lipoprotein cholesterol (HDL-C) were determined on a HITACHI 7450 analyzer (HITACHI, Tokyo, Japan). Low-density lipoprotein cholesterol (LDL-C) was calculated by Friedewald's formula. Fasting plasma glucose concentration (FPG) and 2-hour plasma glucose concentration (2hPG) were measured by the hexokinase method. Serum fasting insulin concentration was measured by electrochemiluminiscence immunoassay (Roche Elecsys Insulin Test, Roche Diagnostics, Mannheim, Germany). HOMA-insulin resistance (HOMA-IR) was calculated by fasting serum insulin (Flns, mU/ml) *fasting blood glucose (FPG, mmol/L)/22.5. HOMA-β was calculated by 20*fasting serum insulin (Flns, mU/ml)/(fasting blood glucose (FPG, mmol/L)-3.5).

### Serum irisin measurement

Serum irisin concentration was measured using the enzyme-linked immunosorbent assay (ELISA) kits (Aviscera Biosciences, Santa Clara, CA). The assay was proven to be highly sensitive to human irisin [12]. The sensitivity of the assay was 0.2 ng/ml and the linear range of the standard was 5 to 500 ng/ml. The intra- and inter- assay variations were both less than 10%.

### Other measurements

Urinary albumin and creatinine were measured on a morning urine sample using an automatic analyser (COBAS INTEGRA 400 plus, Roche, Basel, Switzerland). Creatinine was measured by Jaffe's kinetic method, and albuminuria was measured by immunoturbidimetric methods. Urinary albumin-to-creatinine ratio (ACR, milligram per gram) was calculated. The term of albuminuria was used to describe the increase in ACR of 30 mg/g or over. Serum creatinine (Scr) and uric acid were measured by the autoanalyser (COBAS INTEGRA 400 plus, Roche, Basel, Switzerland). Estimated glomerular filtration rate (eGFR) was calculated using the following estimating equation which was developed by modifying the Modification of Diet in Renal Disease (MDRD) equation based on the data from Chinese CKD patients [16], eGFR (mL/min/1.73 m^2^) = 175×Scr (mg/dL)^−1.234^×age(year)^−0.179^ [female×0.79].

### Definition of metabolic syndrome

According to the International Diabetes Federation (IDF) definitions, for a person to be defined as having MetS, they must have: (1) central obesity (ethnically defined as waist circumference ≥90 cm for Chinese men and ≥80 cm for Chinese women, all of our subjects met this criterion); (2) plus any two of the following four factors: ??? raised blood pressure: systolic BP≥130 or diastolic BP≥85 mmHg, or treatment of previously diagnosed hypertension; ??? raised fasting plasma glucose (FPG)≥100 mg/dL (5.6 mmol/L), or previously diagnosed type 2 diabetes; ??? raised TG level: ≥150 mg/dL (1.7 mmol/L), or specific treatment for this lipid abnormality; ??? reduced HDL cholesterol: <40 mg/dL (1.03 mmol/L) in males and <50 mg/dL (1.29 mmol/L) in females, or specific treatment for this lipid abnormality [17], [18].

### Statistical Analysis

Data were presented as the mean ± standard deviation or median (interquartile range) for continuous variables or number and percentage for categorical variables. Irisin was log-transformed to obtain better approximation of normal distribution. Differences between subjects were analyzed using one way ANOVA or Mann-Whitney U test for continuous variables and chi-square test for categorical variables.

Univariable and stepwise multivariable linear regression was used to estimate the association of clinical characteristics with serum irisin levels. Multivariable logistic regression was used to calculate adjusted odds ratios (OR) and 95% confidence intervals (CI) of raised BP, raised fasting plasma glucose, raised TG level, reduced HDL-C and MetS in different models with adjustment for the potential confounders. In model 1, age and sex were adjusted for; in model 2, educational level, ever smoking and drinking habits and physical activity were further adjusted for; in model 3, SBP, DBP, waist, body fat rate, body muscle mass, albumin-globulin (A/G) ratio, eGFR, ACR, UA, TG, TC, HDL, LDL, fasting glucose, fasting insulin, HOMA-IR, HOMA-β and HbA1c were further adjusted for. P<0.05 was considered significant. Probability criteria to enter or to be excluded from the models in stepwise regressions were set at 0.05 and 0.1, respectively. All statistical analyses were performed using R version 3.0.1 [19].

## Results

Demographic, lifestyle and clinical characteristics of subjects by gender are shown in Table 1. Of the 1,115 participants, 766 (68.7%) were female. The mean age (±SD) of women and men were 53.1 (±7.1) years and 53.3 (±7.6) years (p = 0.702), respectively. Male subjects had higher levels of educational attainment, and were more likely to smoke and drink but less likely to do physical exercise than female subjects. Males had significantly higher levels of BMI (27.8±2.9 vs. 27.3±3.1, p = 0.024), waist circumference (97.1±6.4 vs. 92.1±6.8, p<0.001) and muscle mass (53.9±4.6 vs. 38.3±2.7, p<0.001), but lower levels of body fat rate (26.7±3.3 vs. 38.4±4.2, p<0.001) and serum irisin (median (interquartile range): 5.97(2.55–12.70) vs. 7.78(2.91–15.45), p = 0.024) than females. Men were more likely to be suffered from raised blood pressure (68.2% vs. 51.6%, p<0.001) and raised triglyceride (54.7% vs. 37.0%, p<0.001), but less likely to be suffered from reduced HDL (20.3% vs. 35.6%, p<0.001) when compared with women. There were no statistically significant differences in serum levels of fasting glucose (6.30±1.82 vs. 6.09±1.72), glucose 120-min OGTT (9.06±4.42 vs. 9.05±3.95), fasting insulin (12.4±6.9 vs. 12.9±6.7), HOMA-IR (3.53±2.33 vs. 3.55±2.36), HbA1c (6.28±1.09 vs. 6.18±1.01) and prevalence of raised FPG (61.3% vs. 58.4%) between men and women. The total prevalence rate of MetS was significantly higher in men than in women (69.6% vs. 60.0%, p<0.001).

**Table 1:** Demographic, lifestyle and clinical characteristics of subjects by gender.

Variables	Female	Male	Total	P value
**Demographics**				
N (%)	766 (68.7%)	349 (31.3%)	1115 (100.0%)	
Age (years)	53.1±7.1	53.3±7.6	53.2±7.2	0.702
Education categories, (n, %)				<0.001*
Illiteracy	259 (33.8%)	43 (12.3%)	302 (27.1%)	
Elementary school	214 (27.9%)	116 (33.2%)	330 (29.6%)	
Middle school	155 (20.2%)	101 (28.9%)	256 (23.0%)	
High school	91 (11.9%)	51 (14.6%)	142 (12.7%)	
College or above	47 (6.1%)	38 (10.9%)	85 (7.6%)	
**Life style**				
Ever smoking (n, %)	22 (2.9%)	273 (78.2%)	295 (26.5%)	<0.001‡
Ever drinking (n, %)	10 (1.3%)	120 (34.4%)	130 (11.7%)	<0.001‡
Physical activity (MET-h/week)	112.0 (84.0, 158.2)	46.2 (0.0, 97.2)	84.0 (51.6, 148.4)	<0.001‡
**Clinical characteristics**				
Systolic blood pressure (mmHg)	132.1±18.3	138.1±16.0	134.0±17.9	<0.001‡
Diastolic blood pressure (mmHg)	78.3±10.7	83.2±10.3	79.9±10.8	<0.001‡
BMI (kg/m^2^)	27.3±3.1	27.8±2.9	27.5±3.1	0.024*
Waist circumference (cm)	92.1±6.8	97.1±6.4	93.6±7.1	<0.001‡
Waist hip ratio	0.92±0.05	0.97±0.04	0.93±0.05	<0.001‡
Body fat rate (%)	38.4±4.2	26.7±3.3	34.7±6.7	<0.001‡
Muscle mass (kg)	38.3±2.7	53.9±4.6	43.3±8.1	<0.001‡
A/G ratio	1.87±0.26	2.03±0.32	1.92±0.29	<0.001‡
Triglyceride (mmol/L)	1.69±1.09	2.26±1.57	1.87±1.28	<0.001‡
Total cholesterol (mmol/L)	5.89±1.04	5.81±1.18	5.87±1.09	0.277
HDL-cholesterol (mmol/L)	1.43±0.29	1.22±0.26	1.37±0.30	<0.001‡
LDL-cholesterol (mmol/L)	3.69±0.95	3.57±1.14	3.65±1.01	0.066
Fasting glucose (mmol/L)	6.09±1.72	6.30±1.82	6.16±1.75	0.058
Glucose 120 min OGTT (mmol/L)	9.05±3.95	9.06±4.42	9.05±4.10	0.986
Fasting insulin (mIU/L)	12.9±6.7	12.4±6.9	12.7±6.8	0.292
HOMA-IR (*10^−6^mol*IU*L^−2^)	3.55±2.36	3.53±2.33	3.54±2.35	0.861
Insulin resistance (HOMA-IR > = 2.6, n (%))	461 (60.2%)	206 (59.0%)	667 (59.8%)	0.715
HOMA-β	112.6±59.9	103.8±60.4	109.8±60.2	0.024*
HbA1c	6.18±1.01	6.28±1.09	6.21±1.04	0.124
eGFR (mL/min/1.73 m^2^)	92.7±27.6	90.4±20.5	92.0±25.6	0.163
Reduced renal function (eGFR<60 mL/min/1.73 m^2^, n (%))	29 (3.8%)	11 (3.2%)	40 (3.6%)	0.598
Albumin creatinine ratio (mg/g)	14.9 (9.8, 28.5)	10.9 (7.2, 18.8)	13.8 (8.9, 23.0)	<0.001‡
Albuminuria (ACR>30 mg/g), %	182 (23.8%)	44 (12.6%)	226 (20.3%)	<0.001‡
Blood uric acid (μmol/L)	331.7±77.0	430.1±84.3	362.5±91.6	<0.001‡
Irisin (ng/mL)	7.78 (2.91, 15.45)	5.97 (2.55, 12.70)	7.26 (2.75, 14.72)	0.024*
**Components of Metabolic Syndrome**				
Raised BP (n, %)	395 (51.6%)	238 (68.2%)	633 (56.8%)	<0.001‡
Raised fasting plasma glucose (n, (%))	447 (58.4%)	214 (61.3%)	661 (59.3%)	0.350
Raised triglyceride (n, (%))	283 (37.0%)	191 (54.7%)	474 (42.5%)	<0.001‡
Reduced HDL (n, (%))	273 (35.6%)	71 (20.3%)	344 (30.9%)	<0.001‡
Metabolic syndrome (n, (%))	457 (60.0%)	243 (69.6%)	700 (62.8%)	<0.001‡

*p<0.05,

† p<0.01,

‡ p<0.001.

All percentages are column percentage; except for percentages, all values are mean±s.d. or median (25th, 75th) for non-normal distribution data.

Abbreviations: A/G ratio, albumin/globulin ratio; BMI, body mass index; eGFR, estimated glomerular filtration rate; HDL, high-density lipoprotein cholesterol; HOMA-IR, homeostasis model assessment insulin resistance index; LDL, low-density lipoprotein cholesterol; MET-h/week, metabolic equivalent hours per week.

### Demographic and clinical characteristics stratified by tertiles of serum irisin

Subjects were divided into three groups according to tertiles of serum irisin levels (median (interquartile range): 1.13(0.02–2.73), 7.20(5.48–.00) and 18.70(14.56–.32), respectively). With increasing levels of serum irisin, subjects had significantly less levels of waist circumference, waist hip ratio, albumin-globulin (A/G) ratio and less likely to be ever-smokers. Male gender and muscle mass were negatively associated with circulating irisin level with marginally statistical significance. And there were no statistically significant differences in the levels of systolic BP, diastolic BP, BMI, body fat rate, lipid profiles, FPG, fasting insulin and HbA1c among these three different groups of serum irisin (Table 2).

**Table 2:** Demographic, lifestyle and clinical characteristics of subjects by tertiles of irisin.

Variables	Tertile 1	Tertile 2	Tertile 3	Total	P value
**Demographics**					
N (%)	370 (33.2%)	368 (33.0%)	377 (33.8%)	1115 (100.0%)	
Sex					
Male (n, %)	130 (35.1%)	117 (31.8%)	102 (27.1%)	349 (31.3%)	0.057
Female (n, %)	240 (64.9%)	251 (68.2%)	275 (72.9%)	766 (68.7%)	
Age (years)	52.9±7.5	53.0±7.1	53.7±7.1	53.2±7.2	0.259
Education categories, (n, %)					0.475
Illiteracy	89 (24.1%)	102 (27.7%)	111 (29.4%)	302 (27.1%)	
Elementary school	109 (29.5%)	101 (27.4%)	120 (31.8%)	330 (29.6%)	
Middle school	90 (24.3%)	90 (24.5%)	76 (20.2%)	256 (23.0%)	
High school	48 (13.0%)	50 (13.6%)	44 (11.7%)	142 (12.7%)	
College or above	34 (9.2%)	25 (6.8%)	26 (6.9%)	85 (7.6%)	
**Life style**					
Ever smoking (n, %)	112 (30.3%)	100 (27.2%)	83 (22.0%)	295 (26.5%)	0.035*
Ever drinking (n, %)	46 (12.4%)	44 (12.0%)	40 (10.6%)	130 (11.7%)	0.885
Physical activity (MET-h/week)	84.0 (46.2, 158.2)	84.0 (46.2, 140.0)	98.0 (56.0, 158.2)	84.0 (51.6, 148.4)	0.156
**Clinical characteristics**					
Systolic blood pressure (mmHg)	133.7±17.7	133.9±18.9	134.3±17.0	134.0±17.9	0.904
Diastolic blood pressure (mmHg)	80.3±10.9	79.7±11.6	79.6±10.0	79.9±10.8	0.629
BMI (kg/m^2^)	27.6±3.2	27.4±3.1	27.4±2.9	27.5±3.1	0.688
Waist circumference (cm)	94.4±7.4	93.3±7.0	93.1±6.7	93.6±7.1	0.031*
Waist hip ratio	0.94±0.05	0.93±0.05	0.92±0.05	0.93±0.05	0.002†
Body fat rate (%)	34.4±6.8	34.5±6.8	35.2±6.5	34.7±6.7	0.221
Muscle mass (kg)	44.1±8.7	43.2±7.9	42.6±7.7	43.3±8.1	0.051
A/G ratio	1.99±0.30	1.92±0.28	1.86±0.27	1.92±0.29	<0.001‡
Triglyceride (mmol/L)	1.94±1.37	1.88±1.31	1.79±1.17	1.87±1.28	0.271
Total cholesterol (mmol/L)	5.84±1.07	5.82±1.07	5.94±1.12	5.87±1.09	0.313
HDL-cholesterol (mmol/L)	1.36±0.30	1.35±0.29	1.38±0.29	1.37±0.30	0.524
LDL-cholesterol (mmol/L)	3.60±1.01	3.62±0.99	3.74±1.04	3.65±1.01	0.106
Fasting glucose (mmol/L)	6.21±1.89	6.19±1.91	6.08±1.41	6.16±1.75	0.553
Glucose 120 min OGTT (mmol/L)	9.12±4.20	9.04±4.19	8.99±3.91	9.05±4.10	0.912
Fasting insulin (mIU/L)	13.1±7.2	12.4±6.6	12.7±6.5	12.7±6.8	0.424
HOMA-IR (*10^−6^mol*IU*L^−2^)	3.64±2.34	3.48±2.46	3.51±2.25	3.54±2.35	0.592
Insulin resistance (HOMA-IR> = 2.6, n (%))	227 (61.4%)	213 (57.9%)	227 (60.2%)	667 (59.8%)	0.618
HOMA-β	112.0±61.7	107.3±56.1	110.2±62.6	109.8±60.2	0.566
HbA1c	6.26±1.20	6.19±1.05	6.17±0.85	6.21±1.04	0.460
eGFR (mL/min/1.73 m^2^)	91.7±24.8	94.2±30.2	90.2±20.9	92.0±25.6	0.098
Albumin creatinine ratio (mg/g)	13.5 (8.9, 24.1)	14.0 (8.9, 24.4)	13.8 (8.9, 23.0)	13.8 (8.9, 23.0)	0.840
Blood uric acid (μmol/L)	365.6±89.0	361.5±94.2	360.4±91.6	362.5±91.6	0.721
Irisin (ng/mL)	1.13 (0.02, 2.73)	7.20 (5.48, 9.00)	18.70 (14.56, 27.32)	7.26 (2.75, 14.72)	<0.001‡

*p<0.05,

† p<0.01,

‡ p<0.001.

All percentages are column percentage; except for percentages, all values are mean±s.d. or median(25th, 75th) for non-normal distribution data.

Abbreviations: A/G ratio, albumin/globulin ratio; BMI, body mass index; eGFR, estimated glomerular filtration rate; HDL, high-density lipoprotein cholesterol; HOMA-IR, homeostasis model assessment insulin resistance index; LDL, low-density lipoprotein cholesterol; MET-h/week, metabolic equivalent hours per week.

### Association of clinical characteristics with serum irisin levels

Table 3 shows the associations of clinical characteristics with serum irisin by using the univariable and stepwise multivariable linear regression analyses. In these linear regression models, irisin, as the dependent variable, was log-transformed to obtain better approximation of normal distribution. As for univariable linear regression analysis, similar to the results in Table 2, waist circumference and A/G ratio were negatively associated with serum irisin with statistical significance; while muscle mass and fasting insulin were negatively associated with circulating irisn with marginally statistical significance. After the stepwise multivariable linear regression analysis with adjustment for covariates, fasting insulin, HbA1c and A/G ratio were found to be negatively associated with serum irisin with statistical significance, while waist circumference was negatively associated with irisin level with statistically marginal significance (p = 0.055). BMI, body fat rate, muscle mass, blood pressure, lipid profiles and FPG were not significantly associated with circulating irisin level.

**Table 3:** Univariable and stepwise multivariable linear regression of irisin with clinical characteristics.

Variables	Univariable linear regression			Multivariable linear regression		
	Coefficient	SE	p value	Coefficient	SE	p value
Age	0.0048	0.0093	0.605			
Sex (male v.s. female)	−0.2185	0.1441	0.130			
Educational level	−0.0124	0.0546	0.820			
Ever smoking	−0.2526	0.1515	0.096			
Ever drinking	−0.0545	0.0971	0.575			
Physical activity (MET-h/week)	0.0005	0.0007	0.497			
Systolic blood pressure (mmHg)	−0.0032	0.0037	0.395			
Diastolic blood pressure (mmHg)	−0.0078	0.0062	0.207			
BMI (kg/m^2^)	−0.0258	0.0218	0.237			
Waist circumference (cm)	−0.0231	0.0094	0.014*	−0.0211	0.0109	0.055
Body fat rate (%)	0.0043	0.0099	0.663			
Muscle mass (kg)	−0.0159	0.0083	0.057			
A/G ratio	−1.2588	0.2308	<0.001‡	−1.3376	0.2346	<0.001‡
Triglyceride (mmol/L)	−0.0812	0.0520	0.119	−0.0966	0.0588	0.101
Total cholesterol (mmol/L)	−0.0342	0.0615	0.578			
HDL-cholesterol (mmol/L)	0.0249	0.2266	0.912	−0.4422	0.2582	0.087
LDL-cholesterol (mmol/L)	0.0152	0.0661	0.818			
Fasting glucose (mmol/L)	−0.0242	0.0382	0.527			
Fasting insulin (mIU/L)	−0.0188	0.0099	0.056	−0.0810	0.0332	0.015*
HOMA-IR (*10^−6^mol*IU*L^−2^)	−0.0417	0.0284	0.143			
HOMA-β	−0.0017	0.0011	0.125			
Glucose 120 min OGTT (mmol/L)	−0.0087	0.0163	0.593			
HbA1c	−0.0664	0.0642	0.302	−0.2085	0.1005	0.038*
eGFR (mL/min/1.73 m^2^)	0.0018	0.0026	0.493			
Albumin creatinine ratio (mg/g)	−0.0012	0.0019	0.527			
Blood uric acid (μmol/L)	−0.0011	0.0007	0.143			

*p<0.05,

† p<0.01,

‡ p<0.001.

Irisin was log-transformed to obtain better approximation of normal distribution.

Abbreviations: A/G ratio, albumin/globulin ratio; BMI, body mass index; eGFR, estimated glomerular filtration rate; HDL, high-density lipoprotein cholesterol; HOMA-IR, homeostasis model assessment insulin resistance index; LDL, low-density lipoprotein cholesterol; MET-h/week, metabolic equivalent hours per week.

### Demographic and clinical characteristics by components of MetS

Differences in demographic and clinical characteristics of subjects stratified by raised BP, raised FPG, raised triglyceride, reduced HDL-cholesterol and total MetS are presented in Table 4. Increasing age and male gender were associated with significantly higher prevalence of raised BP and MetS. Lower educational attainment was associated with significantly higher prevalence of raised BP, raised FPG; and ever smoking was positively associated with raised BP and raised triglyceride. Generally, when compared with controls, subjects with raised BP, raised FPG, raised triglyceride and MetS had significantly higher levels of BP, BMI, waist circumference, dyslipidemia, fasting glucose, glucose 120-min OGTT, fasting insulin, HOMA-IR, HbA1c, urinary albumin creatinine ratio, and blood uric acid. Decreasing serum irisin level was significantly associated with higher prevalence of both raised FPG (p = 0.041) and MetS (p = 0.034) but not with either raised BP, raised triglyceride or reduced HDL-cholesterol.

**Table 4:** Demographic, lifestyle and clinical characteristics of subjects by components of metabolic syndrome.

	Raised Blood Pressure			Raised Fasting Glucose			Raised Triglyceride			Reduced HDL-cholesterol			Metabolic Syndrome		
Variables	No	Yes	P value	No	Yes	P value	No	Yes	P value	No	Yes	P value	No	Yes	P value
**Demographics**															
N (%)	482 (43.2%)	633 (56.8%)		454 (40.7%)	661 (59.3%)		641 (57.5%)	474 (42.5%)		771 (69.1%)	344 (30.9%)		415 (37.2%)	700 (62.8%)	
Sex			<0.001‡			0.350			<0.001‡			<0.001‡			0.001‡
Female (n, %)	371 (76.9%)	395 (62.4%)		319 (70.3%)	447 (67.6%)		483 (75.4%)	283 (59.7%)		493 (63.9%)	273 (79.4%)		309 (74.5%)	457 (65.3%)	
Male (n, %)	111 (23.1%)	238 (37.6%)		135 (29.7%)	214 (32.4%)		158 (24.6%)	191 (40.3%)		278 (36.1%)	71 (20.6%)		106 (25.5%)	243 (34.7%)	
Age (years)	51.4±7.1	54.6±7.0	<0.001‡	51.4±7.3	54.4±6.9	<0.001‡	53.0±7.1	53.5±7.4	0.258	53.4±7.2	52.7±7.2	0.137	51.7±7.1	54.1±7.1	<0.001‡
Education categories, (n, %)			0.032*			0.006†			0.094			0.027*			0.248
Illiteracy	113 (23.4%)	189 (29.9%)		103 (22.7%)	199 (30.1%)		182 (28.4%)	120 (25.3%)		198 (25.7%)	104 (30.2%)		102 (24.6%)	200 (28.6%)	
Elementary school	148 (30.7%)	182 (28.8%)		139 (30.6%)	191 (28.9%)		204 (31.8%)	126 (26.6%)		250 (32.4%)	80 (23.3%)		133 (30.1%)	197 (28.1%)	
Middle school	111 (23.0%)	145 (22.9%)		99 (21.8%)	157 (23.8%)		135 (23.1%)	121 (25.5%)		168 (21.8%)	88 (25.6%)		90 (21.7%)	166 (23.7%)	
High school	62 (12.9%)	80 (12.6%)		68 (15.0%)	74 (11.2%)		74 (11.5%)	68 (14.4%)		100 (13.0%)	42 (12.2%)		52 (12.5%)	90 (12.9%)	
College or above	48 (10.0%)	37 (5.9%)		45 (9.9%)	40 (6.1%)		46 (7.2%)	39 (8.2%)		55 (7.1%)	30 (8.7%)		38 (9.2%)	47 (6.7%)	
**Life style**															
Ever smoking (n, %)	100 (20.8%)	195 (30.8%)	<0.001‡	120 (26.4%)	175 (26.5%)	0.987	140 (21.8%)	155 (32.7%)	<0.001‡	239 (31.0%)	56 (16.3%)	<0.001‡	97 (23.4%)	198 (28.3%)	0.072
Ever drinking (n, %)	50 (10.4%)	80 (12.6%)	0.097	46 (10.1%)	84 (12.7%)	0.358	68 (10.6%)	62 (13.1%)	0.011*	116 (15.1%)	14 (4.1%)	<0.001‡	45 (10.8%)	85 (12.1%)	0.400
Physical activity (MET-h/week)	102.2 (56.0, 154.0)	84.0 (46.2, 144.2)	0.054	84.0 (47.4, 148.4)	84.0 (55.1, 148.4)	0.743	102.2 (56.0, 158.2)	84.0 (28.0, 140.0)	<0.001‡	84.0 (46.2, 154.2)	92.8 (56.0, 140.0)	0.363	102.2 (56.0, 156.0)	84.0 (46.2, 144.0)	0.069
**Clinical characteristics**															
Systolic blood pressure (mmHg)	118.5±7.6	145.7±14.1	<0.001‡	129.0±17.3	137.4±17.5	<0.001‡	131.5±18.5	137.2±16.4	<0.001‡	134.1±18.4	133.6±16.7	0.631	122.8±14.0	140.6±16.6	<0.001‡
Diastolic blood pressure (mmHg)	72.0±6.4	85.9±9.6	<0.001‡	77.8±10.8	81.3±10.6	<0.001‡	78.2±11.0	82.1±10.2	<0.001‡	79.7±11.0	80.2±10.4	0.451	73.9±8.8	83.4±10.4	<0.001‡
BMI (kg/m^2^)	26.8±2.7	27.9±3.2	<0.001‡	27.1±2.8	27.6±3.2	0.013*	27.2±3.1	27.8±3.0	0.002†	27.3±2.9	27.9±3.3	0.002†	26.7±2.5	27.9±3.2	<0.001‡
Waist circumference (cm)	91.9±6.1	95.0±7.5	<0.001‡	92.8±6.8	94.2±7.2	0.001‡	92.9±7.1	94.7±7.0	<0.001‡	93.4±7.0	94.1±7.2	0.188	91.7±6.0	94.8±7.4	<0.001‡
Body fat rate (%)	34.8±6.2	34.7±7.1	0.764	34.5±6.4	34.9±7.0	0.275	35.1±6.5	34.2±7.0	0.018*	33.9±6.7	36.6±6.4	<0.001‡	34.2±6.2	35.0±7.0	0.065
A_G ratio	1.93±0.29	1.91±0.28	0.223	1.93±0.29	1.91±0.28	0.288	1.92±0.29	1.93±0.28	0.503	1.94±0.29	1.88±0.28	<0.001‡	1.95±0.29	1.91±0.28	0.020*
Triglyceride (mmol/L)	1.68±1.21	2.02±1.31	<0.001‡	1.70±1.17	2.00±1.34	<0.001‡	1.11±0.34	2.90±1.37	<0.001‡	1.63±1.02	2.41±1.62	<0.001‡	1.23±0.65	2.26±1.41	<0.001‡
Total cholesterol (mmol/L)	5.73±0.99	5.97±1.14	<0.001‡	5.69±1.01	5.99±1.12	<0.001‡	5.64±0.97	6.18±1.16	<0.001‡	6.01±1.04	5.55±1.13	<0.001‡	5.72±0.99	5.96±1.13	<0.001‡
HDL-cholesterol (mmol/L)	1.40±0.30	1.34±0.29	<0.001‡	1.39±0.29	1.35±0.30	0.040*	1.46±0.30	1.24±0.23	<0.001‡	1.48±0.27	1.10±0.14	<0.001‡	1.51±0.29	1.28±0.27	<0.001‡
LDL-cholesterol (mmol/L)	3.57±0.94	3.72±1.06	0.011*	3.53±0.94	3.74±1.05	<0.001‡	3.67±0.88	3.63±1.17	0.470	3.79±0.95	3.36±1.08	<0.001‡	3.66±0.92	3.65±1.06	0.879
Fasting glucose (mmol/L)	5.85±1.39	6.39±1.95	<0.001‡	5.23±0.27	6.79±2.03	<0.001‡	5.96±1.38	6.43±2.12	<0.001‡	6.07±1.61	6.34±2.03	0.018*	5.57±1.19	6.51±1.93	<0.001‡
Glucose 120 min OGTT (mmol/L)	8.15±3.59	9.74±4.33	<0.001‡	7.17±1.80	10.34±4.69	<0.001‡	8.36±3.47	9.98±4.66	<0.001‡	8.66±3.73	9.93±4.71	<0.001‡	7.35±2.73	10.06±4.43	<0.001‡
Fasting insulin (mIU/L)	11.4±5.6	13.7±7.4	<0.001‡	11.0±5.6	13.9±7.3	<0.001‡	11.6±6.3	14.3±7.1	<0.001‡	11.9±6.2	14.6±7.6	<0.001‡	10.0±4.4	14.4±7.4	<0.001‡
HOMA-IR (*10^−6^mol*IU*L^−2^)	3.03±1.75	3.94±2.65	<0.001‡	2.57±1.33	4.22±2.65	<0.001‡	3.15±2.05	4.08±2.61	<0.001‡	3.26±2.00	4.17±2.89	<0.001‡	2.49±1.29	4.17±2.60	<0.001‡
HOMA-β	108.2±55.9	111.1±63.3	0.437	128.9±66.3	96.8±51.7	<0.001‡	102.8±52.0	119.4±68.7	<0.001‡	105.5±58.2	119.6±63.5	<0.001‡	105.0±49.7	112.7±65.5	0.039*
HbA1c	6.05±0.80	6.33±1.18	<0.001‡	5.81±0.34	6.48±1.25	<0.001‡	6.09±0.87	6.37±1.22	<0.001‡	6.15±0.95	6.31±1.21	0.021*	5.91±0.66	6.38±1.18	<0.001‡
eGFR (mL/min/1.73 m^2^)	92.0±28.9	92.0±22.8	0.961	91.8±28.3	92.5±23.6	0.439	92.2±25.2	91.7±26.2	0.765	91.1±24.2	94.0±28.4	0.088	90.9±25.9	92.6±25.4	0.293
Albumin creatinine ratio (mg/g)	11.5 (8.1, 19.4)	15.8 (9.9, 32.7)	<0.001‡	12.2 (8.4, 21.9)	14.8 (9.4, 28.3)	<0.001‡	12.9 (8.5, 22.8)	14.9 (9.5, 29.1)	0.005†	13.2 (8.8, 23.9)	14.7 (9.5, 28.9)	0.023*	11.6 (8.2, 19.7)	15.1 (9.5, 30.4)	<0.001‡
Blood uric acid (μmol/L)	343.1±86.8	377.3±92.4	<0.001‡	352.4±88.5	369.4±93.1	0.002†	343.0±84.2	388.8±94.6	<0.001‡	362.4±92.4	362.5±89.9	0.987	336.6±83.9	377.8±92.5	<0.001‡
Irisin-log transformed (ng/mL)^§^	1.43±1.09	1.19±1.32	0.080	1.46±1.04	1.18±1.35	0.041*	1.38±1.17	1.18±1.30	0.139	1.22±1.31	1.46±1.03	0.099	1.48±1.09	1.19±1.31	0.034*

*p<0.05,

† p<0.01,

‡ p<0.001.

All percentages are column percentage; except for percentages, all values are mean±s.d. or median(25th, 75th) for non-normal distribution data.

Abbreviations: A/G ratio, albumin/globulin ratio; BMI, body mass index; eGFR, estimated glomerular filtration rate; HDL, high-density lipoprotein cholesterol; HOMA-IR, homeostasis model assessment insulin resistance index; LDL, low-density lipoprotein cholesterol; MET-h/week, metabolic equivalent hours per week.

### Associations between serum irisin and components of MetS

Adjusted odds ratios (ORs) with associated 95% confidence interval (CI) of serum irisin for raised BP, raised FPG, raised triglyceride, reduced HDL and MetS are shown in Table 5. In model 1 (adjustment for sex and age), increase of serum irisin was associated with reduced risks of raised FPG and MetS, the adjusted ORs (95%CI) of per standard deviation (SD) increase of log-transformed serum irisin were 0.870 (0.767–0.987, p = 0.031) and 0.872 (0.768–0.992, p = 0.037), respectively. But serum irisin level was not significantly associated with raised BP (OR (95%CI): 0.898(0.792–1.018, p = 0.093)) and raised triglyceride (0.925(0.821–1.043, p = 0.206). In model 2 (additional adjustments for educational level, ever smoking, ever drinking and physical activity) and model 3 (further adjustments for waist circumference, body fat rate, muscle mass, A/G ratio, eGFR, ACR, serum uric acid and all the other potential confounders), the results were quite similar to those in model 1. In the last full model (model 3), the adjusted ORs (95%CI) of per SD increase of log-transformed serum irisin were 0.873 (0.764–0.998, p = 0.046) for raised FPG and 0.796 (0.505–0.959, p = 0.027) for MetS, respectively. Associations of serum irisin with raised BP (0.733(0.454–1.182, p = 0.202)), raised triglyceride (0.954(0.838–1.086, p = 0.478) and reduced HDL-cholesterol (1.130(0.980–1.302, p = 0.092)) were not statistically significant.

**Table 5:** Adjusted odds ratios (ORs) with associated 95% confidence interval (CI) of serum irisin for metabolic syndrome.

	Serum irisin		
Components of metabolic syndrome	OR	95% CI	p value
**Model 1**			
Raised Blood Pressure	0.898	0.792–1.018	0.093
Raised Fasting Glucose	0.870	0.767–.987	0.031*
Raised Triglyceride	0.925	0.821–1.043	0.206
Reduced HDL-cholesterol	1.104	0.966–1.261	0.146
Metabolic Syndrome	0.872	0.768–.992	0.037*
**Model 2**			
Raised Blood Pressure	0.886	0.781–.006	0.063
Raised Fasting Glucose	0.863	0.760–0.981	0.024*
Raised Triglyceride	0.919	0.815–1.037	0.172
Reduced HDL-cholesterol	1.103	0.962–.263	0.159
Metabolic Syndrome	0.863	0.758–0.982	0.025*
**Model 3**			
Raised Blood Pressure	0.733	0.454–1.182	0.202
Raised Fasting Glucose	0.873	0.764–0.998	0.046*
Raised Triglyceride	0.954	0.838–1.086	0.478
Reduced HDL-cholesterol	1.130	0.980–1.302	0.092
Metabolic Syndrome	0.796	0.505–.959	0.027*

Model 1 was adjusted for sex and age;

Model 2 was further adjusted for educational level, ever smoking, ever drinking and physical activity;

Model 3 was further adjusted for SBP, DBP, waist, body fat rate, A/G ratio, eGFR, ACR, UA,muscle mass, TG, TC, HDL, LDL, fasting glucose, fasting insulin, HOMA-IR, HOMA-β, HbA1c;

*p<0.05.

OR and 95%CI was impressed as per SD increase of log transformed irisin.

## Discussion

We found that increasing serum irisin was significantly associated with reduced risks of MetS and raised FPG even after adjustment for potential confounding factors, while the associations of serum irisin with rasied blood pressure and raised triglyceride were not statistically significant. We also found that fasting insulin, HbA1c and serum A/G ratio were negatively associated with serum irisin after adjusting for covariates. Waist circumference was also negatively associated with serum irisin with marginally statistical significance, while there were no significant associations of circulating irisin with BMI, body fat and muscle mass.

Bostrom et al reported that expression of the exercise- and PGC1-α-induced myokine, irisin, drives brown fat-like development of white fat and protects diet-induced obesity and diabetes in mouse models [7]. Irisin is released into blood from skeletal muscle after proteolysis of the type I membrane protein FNDC5, and stimulates uncoupling protein 1 (UCP1) expression and induction of brown adipocytes in white adipose tissue depots, known as white fat “browning” [7]. They also reported that irisin increased total energy expenditure, improved glucose tolerance and reduced fasting insulin in animal models [7]. Testing the association of circulating irisin with insulin resistance conditions in humans, such as MetS and diabetes, may be helpful in elucidating the pathology of these conditions.

Circulating irisin has been found to be reduced in type 2 diabetes patients compared with non-diabetic controls [8]–[10]. Liu and co-workers found significantly lower level of circulating irisin in long-term type 2 diabetes patients compared with non-diabetic controls [8]; and the lower serum irisin was also found in new-onset by Choi et al. [9] and undefined type 2 dibetes patients by Moreno-Navarrete et al., respectively [10]. Because type 2 diabetes and MetS share the same pathology of insulin resistance, it is thus reasonable to speculate the lower serum irisin in MetS patients. However, no evidence was available whether circulating irisin is involved in MetS in adults. The present study found that serum irisin levels were significantly decreased in subjects with MetS and raised FPG than control groups and were independently associated with reduced risks of MetS and raised FPG.

Available evidence about the effects of adiposity and muscle mass on circulating irisin has been controversial. While Timmons JA et al. reported that irisin was not related to BMI in diabetic populations [20], a positive relationship between serum irisin and BMI has been consistently reported in three other studies recently [8], [12], [13]. Huh JY et al. found circulating irisin concentrations were positively correlated to BMI in healthy women [12]. Liu JJ et al. found that, in non-diabetic individuals, circulating irisin correlated positively with age, BMI, total cholesterol, triglycerides, fasting blood glucose, and diastolic blood pressure; and after adjustment for multiple covariates, the positive association of irisin with BMI persisted [8]. Contrastingly, two recent papers reported a negative relationship between irisin level and BMI. Moreno-Navarrete JM et al. found circulating irisin correlated negatively with BMI, waist-hip ratio and fat mass in men [10]. Choi YK et al. recently reported that BMI was negatively correlated with serum irisin, but the negatively association disappeared after the multiple regression analysis [9]. It should be noted that the sample sizes of all of the studies above are quite small, less than 300 subjects. In the present study, with a sample size of more than 1,000 obese adults, we found that waist circumference was negatively associated with serum irisin with marginally statistical significance after the multiple regression analysis; while the negative association of circulating irisin with BMI was not statistically significant. Liu JJ et al. suspected that these discrepancies on the association of serum irisin with adiposity indices may be explained by the difference in their study populations, with some studies focusing on non-diabetes subjects [8,10 12,13], while others focusing on diabetes patients [19]. In the present study with sampling of more than 1,000 obese adults without previously diagnosed common chronic diseases, we failed to find a significant interaction effect of MetS status with adiposity indices on serum irisin in the multiple regression analysis; and the stratified analysis by MetS status found the negative association of waist circumference with serum irisin did not change much (data was not shown). We also suspect that BMI and waist circumference may show different associations with circulating irisin because numerous studies have reported that BMI (a measure of general obesity) and waist circumference (a measure of central obesity or abdominal obesity) have been shown different associations with various health conditions [21]–[23]. Therefore, further studies with larger sample size consisting of different subjects are warranted in future to clarify the association of serum irisin with different adiposity indicators.

Few studies have explored the relationships between irisin and markers of glucose/lipid metabolism, and have showed controversial findings. Timmons JA et al. reported that myocyte expression of irisin was not related to fasting insulin and FPG [19]; while Liu JJ et al. found significantly positive association of serum irisin with FPG but not with other glucose/lipid markers [8]. Choi YK and co-workers found negative correlations of serum irisin level with 2 h plasma glucose (OGTT), HbA1c and triglyceride, but only the negative association of 2 h plasma glucose persisted after multiple regression analysis [9]. In the present study after adjusting for covariates, we found that fasting insulin and HbA1c were negatively associated with serum irisin with significantly statistical significance, and the negative associations of lipid profiles were not statistically significant. Collectively with the data from Choi YK et al., we suggested that decreased serum irisin levels may be associated with insulin resistance and then the development of metabolic syndrome and type 2 diabetes. But only prospective cohort studies with larger sample size and longer follow-up period could clarify this speculation, since the cross-sectional study design of the present study failed to determine the causal pathways of irisin with insulin resistance and MetS.

Interestingly, we found that A/G ratio was negatively associated with serum irisin level which is independent of albumin, globulin per se and other covariates. Further studies need to confirm this finding and elucidate the potential mechanisms underlying.

We must be cautious during interpretation of the present findings due to the following limitations. Firstly, the main limitation of our study is the uncertainty about the temporal sequence among serum irisin, energy metabolism markers and MetS because of the cross-sectional design. Therefore, the results should be confirmed in future prospective cohort and interventional studies. Another limitation is that all subjects recruited into the present study are centrally obese adults, which may hamper us to find more significant differences among different groups and extrapolate our findings to the non-obese adults. Thirdly, we did not measure other cytokines, such as adiponectin, IL-6 and TNF-α, which may be related to serum irisin. These limitations, however, do not diminish the value of this study. For example, our study aims to examining the association of serum irisin with MetS probably with the largest sample size at present. And we have also adjusted for much more potential confounders than previous studies. For example, few previous studies adjusted for physical activity, which was an important confounder of serum irisin [7]–[9].

In conclusion, our study found that serum irisin was independently associated with reduced risks of MetS and raised FPG. In addition, insulin resistance indicators, such as fasting insulin and HbA1c, abdominal adiposity (waist circumference) and serum albumin/globulin ratio were negatively associated circulating irisin level. These results may imply that irisin, a muscle-derived secretory protein, plays a protective role in the pathology of insulin resistance and its related conditions, such as metabolic syndrome and type 2 diabetes. Future work will be needed to determine causal effects of serum irisin on the development of metabolic syndrome and type 2 diabetes in prospective cohort studies and potential benefits of physical exercise on reducing incidences of these insulin resistance related conditions.
